# The Influence of Prism Adaptation on Perceptual and Motor Components of Neglect: A Reply to Saevarsson and Kristjansson

**DOI:** 10.3389/fnhum.2013.00255

**Published:** 2013-06-05

**Authors:** Christopher L. Striemer, James Danckert

**Affiliations:** ^1^Department of Psychology, Grant MacEwan UniversityEdmonton, AB, Canada; ^2^Department of Psychology, University of WaterlooWaterloo, ON, Canada

In a recent opinion paper we argued that prism adaptation (PA) primarily influences motor behaviors and spatial attention in neglect, but may have very little influence on perceptual biases (Striemer and Danckert, 2010b). Furthermore, we also suggested that the effects of PA on motor behaviors and spatial attention in neglect may arise via interactions with the dorsal “vision for action” pathway (Milner and Goodale, 2006), and the “dorsal attention network” that is important for allocating attention to specific locations in space (Corbetta and Shulman, 2002). Thus, we view alterations in shifts of attention following PA as being closely related to changes in motor behaviors (e.g., eye movements) following PA (i.e., the premotor theory of attention; Rizzolatti et al., 1987). See Striemer and Danckert (2010b) for discussion of the effects of PA on attention and motor behaviors and how this may lead to changes in visual imagery tasks.

Support for this hypothesis comes from a recent study (Striemer and Danckert, 2010b) in which we demonstrated that rightward PA reduced neglect patient's rightward bias on a manual line bisection task (i.e., marking the center of a line), but had no influence on their performance on a landmark task (judging whether a bisection marker was closer to the left or right end of a line). These results are consistent with other studies demonstrating that although PA influences exploratory motor behaviors (and covert attention) in neglect, they do not necessarily result in changes in perceptual biases (Dijkerman et al., 2003; Ferber et al., 2003; Sarri et al., 2006, 2010; for a review, see Striemer and Danckert, 2010a). For a similar dissociation between improved attention and bisection performance following PA with no changes in spatial working memory, see Saj et al. (2013).

In a recent opinion paper in *Frontiers in Human Neuroscience*, Saevarsson and Kristjansson (2013) suggest that the results of our recent study are not convincing because both of the tests we used involve “contralesional visual input, as well as eye movements, even when responses were made verbally” (i.e., the landmark task; Saevarsson and Kristjansson, 2013). Saevarsson and Kristjansson also highlight that “difficulties of many patients with shifting their gaze to the contralesional side” may be a critical factor in influencing performance. Based on these criticisms they suggest that the two tests were not capable of isolating “perceptual” and “premotor neglect.”

There are a number of important points to note in reply to these comments. First, we never intended to use the line bisection and landmark tasks to *differentially* assess perceptual and premotor neglect. The purpose of using these tasks was simply to demonstrate that it is *possible* for PA to create beneficial effects for tasks that are completed with the motor effectors involved during adaptation (e.g., a motor response with the adapted hand) without necessarily changing the patient's perceptual bias.

Second, while it is clear that both the line bisection and landmark tasks require contralesional visual input and eye movements, it is unclear how this confounds our interpretation. Specifically, given that the stimuli for both tasks extended into both the left and right visual fields, that patients were allowed unlimited viewing time during both tasks, and that patients were free to make eye movements in both tasks, it is unclear how these factors could have led to the *dissociated* performance we observed (i.e., improvements on the line bisection but not the landmark task). Unfortunately, Saevarsson and Kristjansson (2013) do not construct a plausible alternative account of this dissociation.

Third, while difficulty in shifting gaze contralesionally may be a critical factor in influencing performance, previous studies have demonstrated that, following PA, patients do tend to make many more eye movements into contralesional space (Dijkerman et al., 2003; Ferber et al., 2003; Serino et al., 2006). However, this does not translate into changes in perceptual biases (Dijkerman et al., 2003; Ferber et al., 2003). Again, this provides additional support for our notion that changes in motor performance following PA do not translate into changes in perceptual biases. Of course Saevarsson and Kristjansson (2013) claim that these studies did not properly assess aspects of premotor neglect; however, neither study intended (or claimed) to do so.

Finally, while many studies have isolated the neural correlates of premotor neglect to the frontal lobes and basal ganglia (e.g., Sapir et al., 2007; Rossit et al., 2009a; Vossel et al., 2010), several authors have questioned whether premotor neglect is even an important component of the neglect syndrome (Coulthard et al., 2006; Himmelbach et al., 2007; Rossit et al., 2009b). Specifically, many of the premotor deficits in neglect are also present in right brain damaged patients without neglect. In this sense we view the issue as to whether the patient has been previously diagnosed as having “perceptual” or “premotor” neglect as largely irrelevant to interpreting the validity of our results. The fact of the matter is, regardless of how one attempts to fractionate neglect (in terms of patient diagnosis) it is clear that PA *can* influence motor behaviors *without* influencing perceptual biases (for an alternative view, see Newport and Schenk, 2012).

Saevarsson and Kristjansson (2013) also question our theory suggesting that it is unclear what role the dorsal stream might play in the beneficial effects of PA on premotor neglect. While we agree that it is not entirely clear what brain regions are responsible for the beneficial effects of PA in neglect, or what aspects of PA are critical for generating the beneficial effects (Aimola et al., 2012), it seems undeniable that the very brain regions that are directly involved in adaptation (which prominently include the dorsal stream) must play *some* role in generating the beneficial effects of PA in neglect (see Clower et al., 1996; Danckert et al., 2008; Shiraishi et al., 2008; Luaute et al., 2009; Saj et al., 2013).

In their opinion paper Saevarsson and Kristjansson (2013) proposed that in order for a neglect patient to demonstrate the beneficial effects of prisms they must have either premotor neglect, or a combination of visual neglect and premotor neglect, as visual neglect is assumed to play only a passive role in preventing de-adaptation. In essence, their proposal is quite similar to ours in that they suggest PA is more likely to exert its effects through influencing motor behaviors in neglect, although they focus more specifically on premotor neglect. While there is currently little data available to evaluate the validity of their hypothesis, it is certainly an interesting proposal that warrants future investigation.

In light of this, we are pleased to see that Saevarsson and Kristjansson (2013) and others have taken an active interest in this topic, as any additional knowledge obtained will only serve to help us better understand how PA remediates symptoms of neglect.
